# Developing a Strong Sense of Coherence as a Pathway Beyond Intergenerational Trauma: Narratives of Adult Children of Vietnamese Boat Refugees

**DOI:** 10.3390/ijerph23020266

**Published:** 2026-02-20

**Authors:** Yen Pham, Marguerite Daniel

**Affiliations:** Department of Health Promotion and Development, University of Bergen, 5020 Bergen, Norway; marguerite.daniel@uib.no

**Keywords:** intergenerational trauma, Vietnamese boat refugees, children of refugees, sense of coherence, differentiation of self, salutogenesis, family systems theory

## Abstract

**Highlights:**

**Public health relevance—How does this work relate to a public health issue?**

- Intergenerational trauma is commonly experienced across various populations around the world, making it a public health issue with a burden sustained across generations.
- Understanding how the adult children of Vietnamese boat refugees overcome the intergenerational impact of trauma is essential for a transferrable understanding of adult recovery and movement toward health.

**Public health significance—Why is this work of significance to public health?**

- This study contributes a salutogenic, lifespan-oriented framework for understanding recovery beyond childhood impacts of intergenerational trauma.
- This study shifts the attention from childhood intervention focusing on traumatized parents and child–parent dyads for intergenerational trauma to adult recovery through meaning making, enhanced understanding, and differentiation from maladaptive patterns of childhood family.

**Public health implications—What are the key implications or messages for practitioners, policy makers and/or researchers in public health?**

- Healthcare services for the refugee population should be provided in a culturally responsive manner.
- Seeing the good side of events, drawing meaning from the past, and differentiation from the impacts of childhood trauma-shaped family interactions contribute as potential pathways for adult recovery from intergenerational trauma.

**Abstract:**

Maladaptive family interaction is one of the mechanisms through which trauma is transmitted across generations. The current intervention approach for trauma-affected families focuses on traumatized parents and child–parent dyads during childhood. This leaves a gap in how adult children, who might no longer live with their parents, can overcome the negative impacts of maladaptive childhood interactions with parents as a legacy of parental trauma history. This study focuses on the children of Vietnamese boat refugees in their 30s and 40s in two cities in Norway, applying narrative interviews to elicit long narratives about their lifespan experiences. A hybrid analytic approach utilizes Thematic Network Analysis, informed by a conceptual framework integrating salutogenesis theory and Bowen family systems theory. The findings reveal that maladaptive parent–child interactions in Vietnamese boat refugee families include parents’ high expectations, harsh parenting, children’s obligation to please parents, and adultification, which are trauma-shaped and mediated by Vietnamese culture. Developing a strong sense of coherence (SOC), characterized by enhancing one’s understanding of the self in relation to family, making meaning regarding the past, and playing an active role in reframing relationships with one’s parents, serves as a pathway to outgrow the impacts of maladaptive patterns in one’s family of origin. Overall, this paper contributes a salutogenic, lifespan-oriented framework for understanding recovery beyond childhood impacts of intergenerational trauma.

## 1. Introduction

Trauma exposure is commonly experienced across various populations around the world [1], making it a public mental health issue with multiple layers of influences over individuals, relationships, community, and society [2,3]. This paper focuses on intergenerational trauma, where the influences of trauma are transmitted across generations [4].

Intergenerational trauma refers to a phenomenon in which the impacts of traumatic experiences not only occur in the trauma-exposed generation but also linger on over the next generations [4]. Published studies have shown that the children of trauma-exposed parents are more vulnerable than those of parents without trauma exposure [5,6,7,8]. Refugees who flee to a new country due to safety reasons often experience migration-related trauma, especially before and during their migration journey [9,10]. Previous studies show poor health outcomes among the children of trauma-exposed refugees [11,12]. Notably, refugees’ health status upon arrival in the new country predicts their children’s health outcomes two decades later [13]. This suggests that migration-related trauma has a negative intergenerational impact on refugee families.

Empirical studies on intergenerational trauma among refugee populations suggest that family interaction (i.e., poor parenting) is one of the channels through which trauma is transmitted across generations [4,14]. Most intergenerational trauma interventions focus on parents or parent–child dyads during childhood [15]. The evidence supporting these approaches is enormous. For example, empirical studies focusing on trauma-affected parents address parents’ holistic well-being [16], sense of coherence [17], and social network [12,13,18] as protective factors for children’s health outcomes. Also, empirical studies that focus on parent–child relationships have suggested that adaptive family functioning [19], an improvement in parenting [20], family rapport [5,21], and open communication about past trauma [19,22] could buffer the impact of parental trauma on children. However, there is limited empirical understanding of how adult children can navigate, reinterpret, and heal from childhood trauma-shaped family interactions later in their lives.

The literature refers to the term ‘maladaptive parenting’ [23,24] to describe poor parenting strategies, such as hard discipline and child abuse, which work as a channel of trauma transmission across generations. Inspired by this term, we use the term ‘maladaptive child-parent interaction’ in this paper to recognize the response of children in a poor relationship with their parents. How a maladaptive relationship is defined can be explained by the Interpersonal Acceptance–Rejection Theory [25], which proposes that relationships with significant others, such as parents, that cannot offer individuals warmth, comfort, support, care, and the like may impose a negative impact on their health, sense of self, and worldview.

In this qualitative study, through the perspectives of the adult children of Vietnamese boat refugees in Norway, we explore how intergenerational trauma is experienced in refugee families and how children overcome it. In particular, we first identify maladaptive parent–child interactions shaped by the trauma occurring in a family’s migration history. Next, we evaluate what helps the children overcome the negative impacts of these maladaptive parent–child interactions. As maladaptive parent–child interactions are popularly considered a mechanism of trauma transmission across generations [4], a better understanding of children’s recovery sheds light on intervention directions for the children of refugees.

## 2. Literature Review

### 2.1. Intergenerational Trauma and Relevant Terms

In the broadest meaning, intergenerational trauma refers to the phenomenon that trauma experienced in one generation might impact the health of the subsequent generation [4]. In the literature, the term ‘intergenerational trauma’ is grouped with other relevant terms such as multigenerational trauma, transgenerational trauma, collective trauma, and historic trauma ([26], p. 320) ([27], p. 415) ([28], p. 175). To the broadest extent, all these terms describe the ‘multigenerational nature’ of trauma experienced ([26], p. 320). Accordingly, in the literature, some of these terms are used interchangeably without distinction, e.g., intergenerational trauma and transgenerational trauma [28,29], especially intergenerational trauma and historic trauma [29,30].

However, a slight differentiation has been proposed between intergenerational trauma, collective trauma, and historic trauma. Collective trauma is a massive trauma experienced collectively among a group or community, which could alter the identities, ideas, and interactions within the affected community [29], for example, collective trauma due to floods, hurricanes, storms, eruptions, famine, war, genocide, etc. [31]. Collective trauma, unlike intergenerational trauma, generally refers to the collective effect of trauma within a community or group. Meanwhile, intergenerational trauma often refers to the impact of trauma across familial generations [32,33], emphasizing the intergenerational effect. Examples of intergenerational trauma include studies about the experiences of the descendants of Holocaust survivors [34] or children born of war rape [35]. Historic trauma covers both the collective and intergenerational effects of trauma, that is, trauma is collectively shared among a community or group, and the resultant wounding accumulates or snowballs over time across generations [33,36]. An example of historic trauma is the case of the American Indian/Alaskan Native population; their long history of subjugation through colonization, war, and genocide explains the race-related health disparities between them and the US population [37].

All these forms of trauma can co-occur in refugee populations. Examples of previous studies include collective trauma [38] among Afghan refugees under decades of war and displacement; intergenerational trauma [39] across three generations of Palestinian refugees due to the ongoing impact of settler colonial and military violence; and historic trauma among Southeast Asian refugees arriving in the US [40] due to multiple losses they experienced during a long history of war, political instability, and genocide.

In this study, while the main analytic focus is on intergenerational trauma to describe the effects of trauma across familial generations of Vietnamese boat refugee families, we also address a broader historical and collective context of the community for further understanding.

### 2.2. How Trauma Shapes Parent–Child Interactions

The literature on refugee populations suggests parenting and family relationships are the underlying mechanism that maintains the intergenerational effects of trauma ([4], p. 751). As the focus of this paper, we summarize empirical studies that describe parent–child interaction under the impact of migration-related trauma experienced by refugee parents. Studies that describe intergenerational interaction under the influence of other aspects of refugee families’ experiences, such as acculturation stress and intergenerational cultural gaps, will not be included here.

The parent–child interaction referred to in this article includes the experiences of the parents and the children toward each other, shaped by the impact of the trauma occurring in their family history. Regarding the parental side, the literature on refugee families reports maladaptive parenting among traumatized parents, including violence, hard discipline, high expectations, and overprotection of children. In particular, poor health status (e.g., PTSD symptoms) due to traumatic experiences can impair parents’ parenting capacities [41], which explains why more violence is experienced among the children of traumatized parents than among those of non-traumatized parents [41,42]. Unresolved trauma among refugee parents has been linked to the high expectations they impose on their children, for example, related to education [43,44]. Unresolved trauma also links to parents’ silence about the trauma and the past as strategies to protect their children [45,46].

Regarding the children’s perspective, published studies describe that the children of refugees might feel indebted by their parents’ sacrifice and want to compensate their parents [46]. The literature also describes parentification or adultification among the children of refugees, in which children may take on adult roles in the family [19,47] to take care of their parents emotionally or to be in charge of household demands as a way to alleviate trauma experienced by their parents in the past.

### 2.3. Vietnamese Boat Refugee Families in Norway

During the colonial era, Vietnam was divided into North and South Vietnam. The French transformed South Vietnam toward Western capitalism; meanwhile, North Vietnam followed the road of communism [48]. The cultural difference between the two regions continued during the American sponsorship period, after French colonization. When communism seized control over the country, which led to the Fall of Saigon [49], a substantial population in South Vietnam fled the country [48].

Vietnamese boat refugees, or the boat people, refer to those who escaped Vietnam by boat ([50], p. 84). An estimated 200,000–400,000 boat people did not survive storms, overcrowded boats, and dangers from pirates on the sea, and were not able to receive humanitarian help to assist their access to transit camps en route to recipient countries [50].

According to Statistics Norway ([51], p. 26), more than 10,000 Vietnamese people came to Norway as refugees. Of these, more than 6000 were main refugees, while the rest, more than 3000, came through family reunification with main refugees. Resonating with the experiences of other refugee groups, Vietnamese boat refugees also experienced hardship before and during their migration journey. Published studies describe the traumatic experiences of Vietnamese refugees when they were in Vietnam, such as witnessing deaths and severe injuries from war, torture, and military experiences [52]. After fleeing from Vietnam, refugees experienced challenges, including waiting in refugee camps, being separated from their families, suffering injuries, witnessing the deaths of family members, experiencing violence, and being assaulted [53]. At the family level, these historical traumas of war and displacement have negative impacts on the mental health of refugees [54], which then impacts their children’s mental health [13].

A group of studies in Norway follows up on Vietnamese refugees who arrived in Norway from transit camps after two decades, describing their difficulties in adapting to their new country, such as poor participation in the workforce [55] and acculturative hassles [56] related to learning and using the Norwegian language. In this context, Vietnamese refugees relied on the Vietnamese community in their new country to help them raise their children and adapt to the new country [57]. The existing literature also describes the potential conflicts between parents and children due to cultural gaps in the new country [58].

## 3. Theoretical Framework

### How Children Can Overcome the Intergenerational Impact of Trauma

How can the children of Vietnamese boat refugees overcome negative impacts from the maladaptive parent–child interactions as the legacy of their family’s migration-related trauma? In seeking a theoretical framework of how one grows beyond the negative impact of their family, we engage in the Bowen family systems theory [59,60] and Antonovsky’s theory of sense of coherence ([61], p. vii), ([62], p. 19).

According to Bowen, a family functions like a whole system, in which every part is interconnected and influences the other ([59], p. 351). Under the impact of the family system, the child develops with varying levels of self on a continuum, from a confusion of self to a differentiation of self ([60], pp. 108–109). Confusion of self is an ill state, referring to circumstances when one is not able to develop their unique self, so their lives and choices are passively directed by their family’s impact, which helps repeat the patterns of the family system ([60], pp. 108–109). Meanwhile, differentiation of self is a mature state, referring to circumstances when one develops a well-defined self, so that one can direct one’s own life and choices beyond the family system’s impact ([60], pp. 108–109).

To understand the intergenerational transmission of trauma among refugee families, according to this theory, when parents are affected by migration-related trauma, their children are affected throughout the interconnections and interactions within the family system. These maladaptive parent–child interactions might impact children and be detrimental to their health. When the children are passively affected by the impact of trauma transmitted through family interactions, they have a ‘confusion of self’, mirroring the dysfunctional family system. However, if the children can overcome the impact of trauma transmitted through maladaptive family interactions, they develop a differentiated self, indicating their ability to separate themselves from the dysfunctional family system, to grow beyond it, and be able to direct their own lives and choices.

But how can one shift from being influenced by maladaptive family interactions to acquiring a differentiated self? The lens of health promotion considers this mobilization from illness (confusion of self) toward health and well-being (differentiation of self) as a process of enhancing a sense of coherence, facilitated by generalized resistance resources [62], ([63], pp. 95–99). A strong sense of coherence (SOC) is an indicator of well-functioning and healthy individuals, characterized by three dimensions, including the *cognitive* aspect (comprehensibility)—the belief that challenges are understood and that the internal and external worlds are consistent, structured, and clear, rather than chaotic or disordered ([62], p. 17); the *behavioral* aspect (manageability)—the belief that one has available and adequate resources to cope with a challenge in a salutary manner [64]; and the *motivational* aspect (meaningfulness)—‘a way of looking at life as worth living, of seeing stressors as perhaps painful and yet worthy of being coped with rather than anaesthetized’ ([65], p. 79). In empirical studies, many interventions measure SOC as an indicator of health improvement [66,67,68]. SOC also shows an alignment with many other well-known health-orienting indicators, such as quality of life and self-esteem [68]. Next, generalized resistance resources (GRRs) are ‘any characteristic of the person, the group, or the environment that can facilitate effective tension management.’ ([69], p. 99). According to Antonovsky, GRRs facilitate the development of a strong SOC ([63], pp. 95–97).

While the family systems theory helps to address a solution allowing one to outgrow the intergenerational impact of trauma transmitted through the family system, it falls short in addressing how the mobilization toward this well-being state (differentiation of self) from illness (confusion of self) occurs. The combination of the Bowen family systems theory and Antonovsky’s framework enables us to cover both aspects—the outcomes and the pathways. In particular, we adopt the general three-component structure of SOC and apply it to describe a differentiated self. That is, the development of a differentiated self is interpreted as an SOC-enhancing process, facilitated by GRRs, characterized by the enhancement in *comprehensibility*—the ability to reflect on themselves in relation with their family relations and develop a thorough understanding about how family situations and how family interactions impact themselves; *manageability*—the ability to use resources to play an active role in relationships with parents and to configure their own choices and lives apart from family impacts; and *meaningfulness*—insights or meaningful lessons learned from experiences with family. What is more, this combination of two frameworks is appropriate since SOC’s sub-components are strongly aligned with the psychological standpoint on interventions for children of traumatized parents—emphasizing the importance of one’s ability to reflect and understand oneself in relation to significant others in the family. A thorough understanding and insights are the key to helping one play an active role in these relationships and break the trauma cycle transmitted through family interactions ([70], p. 97), [71,72].

This understanding of the development of a strong sense of coherence (a differentiated self) as a pathway to grow beyond the impact of trauma transmitted through maladaptive family interactions will guide our data analysis for this paper.

## 4. Methods

This paper is a part of a larger qualitative study. The original sample of participants includes six adult children of Vietnamese boat refugees and four religious leaders, as presented in Table 1. Participants were recruited through gatekeepers, as well as the snowball and convenience strategies, in Oslo and Bergen from June to October 2023. The first author reached out to Vietnamese grocery stores and religious centers to inquire whether they are aware of any children of boat people (‘con của thuyền nhân’). The children of Vietnamese boat refugees had to meet the inclusion criteria of being the first child born to Vietnamese boat refugee(s) after their parents arrived in Norway or having come along with their parents when they were little to ensure that they were not immediately exposed to migration-related trauma. For triangulation ([73], p. 66), Group 2 participants are religious leaders who have served the Vietnamese community in Norway. The religious leaders were recruited through Catholic churches and Buddhist temples, since these are the two main religious affiliations of the Vietnamese community in Norway ([74], p. 3).

The narrative interview [75] was used to elicit a long, narrative response. The adult children (Group 1) were asked three main broad questions: “Could you tell me about your parents’ experiences when they left Vietnam and moved to Norway?”; “Could you tell me about your life?”, and “In your opinion, how do your parents’ experiences influence your life experiences?”. For each question, the first author then used the prompts to help the participants navigate their stories: stages (before leaving Vietnam, during the journey, after arriving in Norway), timeframe (childhood, teenager, etc.), location (at home, at school, at work, etc.), and events (difficulties, pathways to overcome). Similarly, the religious leaders (Group 2) were asked about their observation of the experiences of children of Vietnamese boat refugees, focusing on the refugee parents’ experiences, the children’s experiences, and how the refugees’ experiences influenced their children’s experiences. The average interview duration was one hour, ranging from 40 min to one and a half hours. Interviews took place at workplaces, cafeterias, or homes based on participants’ convenience and preferences. Before the data collection, the proposal for data processing and management was submitted to SIKT (The Norwegian Agency for Shared Services in Education and Research) for an ethical assessment. All participants were informed about the research aim, how data would be processed, and their rights in terms of voluntary participation, confidentiality, and anonymity or partial anonymity (religious leaders). Ten participants agreed to participate in this study.

Due to the narrative interview method, rich and extensive data were collected about the experiences of children of Vietnamese boat refugees, highlighting two aspects: acculturation and intergenerational trauma. On the whole, religious leaders described their observations of the former aspect rather than the latter. For the interest of this paper, the religious leaders were excluded from the data analysis for this paper. One participant (Nhi), who left Vietnam at the age of five years old, was also excluded from the analysis for this paper, as this person might have been directly exposed to migration-related trauma, which makes her experience with trauma different from the other participants. The findings reported in this study are hence based on data from five participants, Hung, Huy, Hieu, Hau, and Ngoc, as shown in Table 1.

Data analysis was conducted following Thematic Network Analysis [76]. While this analysis shares key features with other qualitative analyses in uncovering themes from both implicit and explicit statements, its strength lies in offering an organizing principle and a presentational means ([76], p. 388). The step-by-step process of labeling codes and constructing basic themes, organizing themes, and global themes is systematic, rigorous, and transparent. The interviews were transcribed and managed using NVivo 12. The first author read the transcripts line by line to generate codes that are relevant to our excerpts. Related codes were grouped into basic themes, which were then combined into organizing themes. Then, organizing themes were clustered to construct global themes, as presented in Table 2 and Table 3. While the process of generating codes and lower themes (basic themes) is inductive, the process of constructing higher themes (organizing themes, global themes) is deductively guided by the theoretical framework. Given the rich and extensive narrative data, using a hybrid analysis approach ensures a thorough understanding of narratives while enabling well-framed findings.

The study’s credibility, which deals with the congruency of the findings with the researched realities, is handled as follows: obtaining thick descriptions and concrete details of the researched phenomenon ([73], p. 64) through applying a narrative interview strategy, spending considerable amounts of time getting along with the Vietnamese communities in Norway, and applying triangulation, in terms of using different types of information sources [73], p. 66), to contrast and compare the collected data from different groups.

## 5. Findings

The findings describe maladaptive parent–child interactions as the manifestations of intergenerational trauma occurring in Vietnamese boat refugee families, which is followed by pathways through which the children grow beyond these family impacts.

### 5.1. Intergenerational Trauma Manifested Through Parent–Child Interactions

Two types of maladaptive parent–child interactions were revealed in Vietnamese boat refugees. The first type, on the parental side, was parents imposing high expectations on their children to succeed in school and practicing harsh parenting. The second type, on the children’s side, was feeling obligated to please their parents as compensation for parental hardships, or carrying out adult roles from an early age to share family responsibilities with their parents. The children of Vietnamese boat refugees in this study experienced either of these two types of maladaptive relationships with parents.

#### 5.1.1. Maladaptive Parent–Child Interactions

The first type of maladaptive parent–child interaction is poor parenting. The children of Vietnamese boat refugees (Huy, Hau, Ngoc) described that their parents imposed high expectations on them related to school success. In particular, the refugee parents expected their children to focus on studying exclusively and did not allow their children to have interests or hobbies. As Huy recounted:

*‘At that time, I did not remember exactly what he [his father] talked about. I think he wanted me to focus on studying … I was just six or seven years old at that time …If I hung out with my friends, or as soon as I touched the ball [to play football], my father scolded me.’*

Hau also echoed Huy’s story that *‘If I don’t do homework, if I don’t go to school, or if there are any troubles at school, my parents punish me harder … I meant physical punishment’.* Huy, Ngoc, and Hau also described that they got beaten a lot when they were little at home. Huy recounted the parenting he experienced at home as a child:

*‘I could not figure out anything that I did that could satisfy my father. I hung out with my friends, I came back home late by five minutes, and I got beaten. I caused a fight at school with those who bullied my brother, and I got beaten. I got reported to my parents about issues at school, and I got beaten. My friends and I collected bottles to trade for cash to buy candies. My father found the candy wrappers, and I got beaten … [In adulthood] He commented on everything I did—making friends, girlfriends, clothing style, bearing style, everything.’*

Especially since Huy tried to play football secretly during his childhood without his father’s permission, he was always afraid that his father would find out, which would cause serious punishment. However, his father finally found out and continued to prohibit him from playing football, even when Huy was selected by the national football club. As a reflection in adulthood, Huy said that the only way to please his father so that his father would let him play football was his excellent achievements in school. As Huy recounted:

*‘At 12 years old … when I climbed the fence [after joining the football club], my head, every day, three times per week, was always repeating ‘Dad doesn’t pass by, Dad doesn’t pass by, Dad doesn’t pass by’—My mind kept repeating. Having climbed the fence without being seen, how relieved, how scary… When I was 15 years old, that day, […] when I was just putting one leg on the fence, my dad passed by. That day, when I got back home, my dad did not scold me, but I had already cried … One day, when I was playing for my club team, I saw him watching me from the fence […]. I did not understand why he came. In my head, I thought he came to claim that I was useless […] so he would ban me from playing football […] Before seeing him, I had scored three goals, […] but since I saw him […] I could not play anymore […] When I was nearly 17 years old, I was chosen as a main player of a football club […] they wanted to sign the contract with me […], but my dad did not want me to play football. I was not old enough to sign the contract […]. Since then, I had pinned my hatred on him. I hated him so much […] That pressure is unsolvable. […] I think if I could come back to my thirteen and fourteen, I could advise myself that, okay, I should have pleased my father’s intention, then he would have […] let me do something that I was not allowed to do back then. […] Maybe, at 13 and 14 years old, had I obeyed him not to play football for one or two years and stayed focused on studying, always gotten the highest score at school … my father would have rewarded me with something to make me happy. Then, when I was 16 … [he would have signed the football contract for me].’*

The second type of maladaptive parent–child interaction revealed in this study is the children’s obligation to compensate their parents for their migration-related difficulties in the past. The children would try to please their parents or carry out adult roles in the family from an early age. As Hieu noted, he always tried to please his parents, which caused him stress:

*‘I’m the oldest grandson on my grandfather’s side, … I had some pressure on myself to become successful, so that’s why … I chose to become an architect because an architect in Vietnam, for us, is a high-standard profession. Nobody on my father’s side had ever had higher education, so my parents were very happy and proud of that … It was hard work … I was giving my parents something to be happy about, and they went to tell other Vietnamese families that I was this and I was that…’*

Hieu explained that he knew his parents had worked so hard for him to have this life and that he could not just waste it away.

Hung’s story also resonates with Hieu’s story in the way he took care of his parents. Since an early age, Hung carried out numerous family responsibilities, including providing translations, preparing tax documents, and negotiating with a lawyer and with the house-building contractor for his parents. Also, as a child, Hung was sensitive to his parents’ emotions. Hung noticed when his parents were stressed: ‘*I knew my parents were stressed even though they did not tell us, the children. I stayed under the same roof with them, so I just knew, I listened, and I knew*.’ Notably, he even sacrificed his education to support his family, as Hung described: ‘*It was a difficult time, so I thought I could do education later. I decided to go to work to reduce my parents’ financial stress*.’

#### 5.1.2. Children’s Emotional Confusion in Childhood Families

These maladaptive parent–child interactions create emotional confusion for the children, obstructing them from developing an adequate understanding of themselves (i.e., acknowledging their genuine choices and capabilities), as well as their ability to navigate their relationships with their parents. For Huy, Hau, and Ngoc, who experienced poor parenting by their parents, as children, they experienced emotional confusion about their parents. In particular, Huy found that regardless of whatever he did, he was never adequate to his father. Similarly, as a child, Ngoc found her parents’ high expectations *‘weird’* and *‘unfair’*:

*‘the expectation is like … unfair because they haven’t gone to school, so they don’t know how difficulties … So, they have like really weird expectations, that everything is easy, and everything should be easy because here [it] is a free country, school is free, you know … everything is free, so everyone can be whatever they want. Why they didn’t have anything, they still got everything. If they can do this, of course, we can do it.’*

She also found her childhood home a *‘chaotic’* place where she and her siblings got beaten a lot. Likewise, as a child, Hau felt embarrassed about his parents when he compared himself with his classmates, since he was the only one who came to class with bruises from his parents’ physical punishment at home: *“I had a lot of friends that were Norwegian … we’re always comparing [sharing about experiences at home] … only me coming to school with bruises”*.

For Hung and Hieu, who, as children, tried to compensate for their parents’ migration-related difficulties, they experienced emotional confusion about themselves. Since Hieu tried to please his parents and meet their expectations, as a child, he was confused about his own capabilities. As Hieu articulated:

*‘I didn’t feel, sometimes, … I am not good enough. I always put up a façade to keep my Vietnamese parents happy, and make them feel good, while me, … I know that I was not perfect and maybe not as smart and right as they think I was.’*

This doubt increased when he was pursuing higher education in the profession of architecture, and even more after two consecutive family losses, which affected his academic performance. Hieu felt worst about himself for being an underachiever:

*‘I felt ashamed, I felt that I was a loser, … like I didn’t deserve to be there, among other people who worked really hard to get good results. I also worked very hard, but I didn’t get any results … I was feeling ashamed, and it went so far that I got social anxiety, because I felt like I didn’t belong there.’*

In Hung’s story, he did not clearly mention that the way he carried out adult responsibilities as a child imposed a negative impact on him, but he wished he had been able to continue his higher education after his family’s financial pressures were solved—in fact, he did not. As Hung described: ‘*If I could go back, I’d be determined to go to university, then my path would be easier. I feel that decision [sacrificing his education to support family] was somewhat wrong*.’

### 5.2. Developing a Strong Sense of Coherence to Grow Beyond the Intergenerational Impact of Trauma in Refugee Families

In this study, the pathway through which the children of Vietnamese boat refugees grow beyond the intergenerational impacts of trauma characterizes the development of a differentiated self, as presented in Table 3. In particular, the shift to a differentiated self, equivalent to a sense-of-coherence-enhancing process, was facilitated by social support, health services, improvements in the parenting of refugee parents, and a temporary distance from parental impacts in their adulthood. The children show an enhanced understanding of themselves in relation to their families and their parents’ migration-related difficulties; they were able to reframe their connections with their parents, in which they could direct their own lives and choices free from their parents’ impacts; they could perceive their parents as a source of peace and inspiration, instead of a source of stress like they had experienced as children.

#### 5.2.1. Resources for the Development of a Strong Sense of Coherence

The shift from being affected by the maladaptive parent–child relationship to developing a differentiated self, a sense-of-coherence-enhancing process, starts with a temporary distance from the family’s impact, which was experienced by all the adult children. The first type of temporary distance occurred due to the children’s effort to avoid their parents. For example, Hau and Ngoc moved out to be away from their parents once they reached adulthood. Huy tried to avoid his father by finding a job far away from home. Physical distance as a means of avoidance is common among those children who experienced strict parental discipline in childhood. For example, Huy described his avoidance of his father:

*‘Every day after school, I stopped by my Mom’s shop and asked my Mom if she was doing well […] However, whenever my Dad came, I left. I did not want to talk to my Dad. […] Regardless of what I did, he always opposed … Up to a certain point, I had to leave, to work on what I want.’*

The second type of temporary distance occurred thanks to the natural transition into adulthood. For example, Hung got married and invested more in his own new family. Hieu waited for graduation from the architectural university, which marked his completion of the family’s wishes, to have his own freedom.

*‘And then I graduated, it was a very good relief. From that moment, I want to … yeah, to live my life. […] After turning to 30s, I don’t value it [school achievements] as much as personal freedom, and being happy and feeling optimal and having a good emotional life.’*

Temporary distance due to this natural transition is common among those children who, in childhood, felt obligated to please their parents or sacrifice for their parents.

The analysis also reveals other resources that facilitate the development of a differentiated self, experienced by the children of Vietnamese boat refugees. The first resource is social support. Ngoc described that when she was trying to avoid her parents during childhood, she leaned on her siblings, which maintained her feelings of family. Hieu also mentioned the support of his friends and teachers in helping him confront his anxiety: ‘*After an event at school, I found out what happened to me in the confrontation … So, I confronted and told them how it was, and then they … the teachers were starting to believe me’*. The second resource is health services. Particularly, Huy mentioned the important role of a psychologist in helping him understand the relationship between himself and his father. Also, reflection and psychological confrontation are psychological strategies that Hieu and Ngoc applied to understand themselves in relation to their families. The third resource is a change in the parents. In Huy’s story, his father changed when Huy was nearly 40 years old, which finally eased his struggle in his relationship with his father. As Huy recounted:

*‘In the recent five years, I could feel at ease. He [his father] called and asked me if I was doing well, and how I played football as a child, since he found a lot of medals at home … That day, when I received a call from him, I felt like I had never been as happy as I was that day … I felt like 100 kg lifted off my shoulders … That is to say, no pressure in my life could compare to the pressure my father imposed on me.’*

#### 5.2.2. Enhancing Understanding of Self in Relation to Families

The children of Vietnamese boat refugees developed a more thorough understanding of themselves in relationships with their parents in their 30s and 40s. They understand how migration-related trauma impacted their parents’ parenting (Ngoc), how their parents’ poor parenting impacted their experiences (Huy), as well as how carrying out the obligation to please their parents caused them stress (Hieu). For example, Ngoc described her understanding of how her parents’ migration-related trauma translated into her parents’ high expectations for her and her siblings:

*‘They lost their culture, they lost the language, they lost the land, they lost the family, … That’s not easy, they kind of tried to live through the kids, … wanting, wishing, … forcing us to a path we can succeed …’*

Also, Ngoc understood why her parents applied strict discipline to her and her siblings:

*‘They lost everything … All they got were us, and we just tried to integrate into Norway, that is the biggest loss… They did it [bad control] ‘cause that’s the only way they know, that’s the only way they can. Can imagine the frustration that the kids, eleven kids that are the only ones you have, then turn their backs on you because they don’t want to speak the language, they don’t really want to keep the culture, and they just want to be a part of, you know, the Norwegian culture.’*

Huy, who experienced his father’s hard discipline, gained an understanding of how the negative impacts of his relationship with his father translated into his relationships with others. Huy recounted that he had to use psychological support in his adulthood since he could not control his anger toward others:

*‘The psychologist helps me to understand what makes me get angry. Regardless of how excellent I am in football or at work, I still have something repressed inside. I was under high pressure from my father, who made me feel I was never good enough. This way, I also apply high demands to others.’*

Hieu, who tried to please his parents from an early age, also described his understanding of how keeping the façade to please his parents made him ill. Later on, this burden was lifted off his shoulders through a psychological confrontation at school, thanks to the support of the teacher and friends. He confronted and told his teacher and classmates about what causes his anxiety. This confrontation marks his clear understanding of himself in relation to his parents and family situation.

#### 5.2.3. Reframe the Position of Self in Relation to Family

A thorough understanding of the maladaptive interactions with parents in the past helped the children of Vietnamese boat refugees in this study reposition themselves in these interactions. Those who avoided their parents due to their parents’ poor parenting are now able to reconnect with their parents (Huy, Ngoc, Hau). For example, Huy no longer becomes angry at the presence of his father; he decided to forgive him, and he was then able to have dinner with his father and talk about his achievements in football in the past: *‘My father has now retired for 20 years … when I come back home for family meals, I recognize his regrets so I don’t blame him anymore. I want to be closer to him’*. Likewise, Ngoc, after 15 years living in another city to avoid her childhood family, could reconnect with her parents and enjoy food as her parents’ *‘language of love’*, instead of seeing home as a bad and sad place, as she articulated:

*‘[…] when I was growing up in my house, my parents, because of the situation, we just saw the sad part […], the way of acting, the way of showing love, that was just their ways from that time […]. Now I love them to death. Just a period in my upbringing that I hated them [my parents] as so many kids do.’*

Those who lost themselves due to always trying to please their parents or carry out household responsibilities are now able to follow their own interests and leave behind the obligatory tendency to please their parents. For example, after graduating with a degree in architecture, Hieu pursued his interest in dance and became a known dancer in Europe. Likewise, Hung, who took an adult role in childhood, is now taking care of his own family and lets his sister help with taking care of his parents.

#### 5.2.4. Experience Families as a Source of Ease and Inspiration

At the time of the interview, in their 30s and 40s, all the adult children were able to experience their relationship with their refugee parents as a source of ease and inspiration. Those who felt *‘embarrassed’* (Hau) of, *‘pinned a hatred’* (Huy) on, or *‘hated’* (Ngoc) their parents at an early age could feel calmer and at ease with their parents. They acknowledged more positive sides of their families, which they could not see as children (Hau, Ngoc). Those who lost themselves when they tried to please their parents (Hieu) or sacrificed their own interests for their parents (Hung) have now gained a better sense of themselves and their own lives (Hieu, Hung). In particular, most of the adult children referred to their parents’ migration-related trauma as a source of inspiration for them in dealing with difficulties in their lives. As Ngoc described:

*‘You are a mom yourself, you’ve gotten two children, and you know, … how hard it is to raise two children. So just imagine, how it is to have like 11 kids … how it is to not speak the language, not have money or anything … My Mom and Dad didn’t complain that much [though] they had more problems than me … Then I faced it at work. I solved all the wrong things [in my life].’*

## 6. Discussion

This paper echoes prior studies regarding the motivation of trauma-shaped child–parent interactions, including parents’ high expectations (e.g., [43]), strict parenting (e.g., [42]), children’s obligation to please parents (e.g., [46]), and children’s adultification (e.g., [47]). However, these manifestations of intergenerational trauma might be slightly mediated by the culture. For example, what might be unique in the experiences of the children of Vietnamese boat refugees is that nearly all these maladaptive interactions in this study are likely related to the Vietnamese parents’ attitudes toward the value of academic success. That is the reason why, when the children navigated how to please their parents, they chose to be excellent at school or enroll in the profession perceived as most prestigious by the Vietnamese community (i.e., Hieu’s narrative). The trauma-affected parents protected their children from hardships by forcing them to succeed at school and applying strict parenting when the children did not focus on studying. Huy could not find anything he did that could satisfy his father, yet he tried to be excellent at school, which was also his insight later on in adulthood. The parents’ obsession with their children’s school success might cause constant parent–child tension, given that children engage in education for at least 12 years during their childhood in Norway. Accordingly, in supporting refugee families, it is worth considering the culturally shaped motivations behind parents’ maladaptive parenting, instead of just trying to change them.

If manifestations of trauma-shaped child–parent interactions are mediated by the culture they belong to, how much are these interactions observed in that culture when the intergenerational impact of trauma is not present? While the manifestation of intergenerational trauma might sound like it just reflects the values and practices of Vietnamese families, regardless of trauma effects, such as strict parenting or high achievement in school [77], the motivation behind them might be different between those with and without trauma effects. First, although Vietnamese children are culturally encouraged to obtain high academic achievements [78], one of our participants (Hieu) chose to study hard as a way to please his parents to compensate for their hardships. This motivation of compensating parents for their hardships echoes other trauma-affected populations [46]. Next, an additional motivation behind the strict parenting or high academic achievement expectations experienced by the participants might be parents’ unresolved trauma or strategies to protect their children from hardships. This trauma-driven parenting caused great confusion for our participants in their relationship with their parents. In other words, while the nature of child–parent interactions is driven by both intergenerational trauma effects and Vietnamese cultural values, a probable sign to differentiate between those with and without trauma effects within the same cultural context is whether the children experience great confusion in their relationship with their parents and about themselves.

Despite experiencing maladaptive interactions with refugee parents in childhood as a legacy of war and migration-related trauma in family history, the adult children of Vietnamese boat refugees could overcome the intergenerational impact of trauma through developing a strong sense of coherence.

How does the development of a sense of coherence help individuals overcome the intergenerational impact of trauma? Through applying the Bowen Family Systems framework [59,60], this paper addresses that developing a differentiated self is the pathway to outgrowing the negative impacts of one’s family, instead of maintaining a confused self where individuals continue being passively directed by family impacts, which serves as a mechanism of repeating trauma-impacted patterns across generations. Furthermore, the shift from a confused self (ill state) in childhood to a differentiated self (with better health and well-being) is equivalent to the process of enhancing a sense of coherence, facilitated by generalized resistance resources (GRRs). Indeed, this study contributes a salutogenic, lifespan-oriented pathway for understanding recovery from intergenerational trauma.

Interestingly, the shift to a stronger sense of coherence among all the adult children in this study started first and foremost with a temporary distance from their parents, either by an intentional move out of the childhood home, finding a job far away from home, or through the natural transition to adulthood due to having their own family or completing their parents’ wishes. This temporary distance from maladaptive interactions with parents is important, giving the adult children space to recognize what is truly meaningful to themselves and their own lives, apart from parents’ expectations or obligations. Among those who experienced strict parenting, physical distance could help them feel calmer and safer in sorting out their lives, which resonates with the experiences of some participants in a prior study ([46], p. 418). Furthermore, looking at this from Antonovsky’s understanding of coping [61], temporary distance likely helps children avoid facing stressors that establish a tension state, where the challenge is still present, but children gain more manageability and control over the situation.

Next, social support plays an important role in helping children deal with the intergenerational impact of trauma. When the children could not find peace in their relationship with their parents, who could they lean on? This is when support from, for example, siblings, friends, and teachers becomes very important. These social resources appear in the adult children’s narratives as individuals they could trust and to whom they could reveal and confront their vulnerabilities (e.g., Hieu’s story) or lean on when their relationship with their parents was a source of chaos and stress (e.g., Ngoc’s account). This is aligned with Antonovsky’s notion of ‘legitimate others’ as a resource for health ([62], pp. 17–18).

However, in one case in this study, temporary distance and social support were not enough to overcome the intergenerational impact of trauma. Huy could not resolve his struggles in his relationship with his parents until his father called him to ask how he was doing and showed recognition of his passion for football in the past. This suggests that when the root cause of low-quality parent–child interaction lies with the parents, a change in the parents may be very important for the health and well-being of the children. This also aligned with the current intervention approach to intergenerational trauma [15]—improving parenting and the health conditions of traumatized parents.

It is not easy to address which of the three SOC components is the most important for the children of Vietnamese boat refugees in overcoming the intergenerational impact of trauma. According to Antonovsky ([62], p. 22), meaningfulness is the most crucial component, serving as a sense of motivation for an individual to see their challenge as worth coping with. However, for the adult children of Vietnamese boat refugees in this study, some of them avoided their parents for nearly two decades, without any motivation to reconnect (e.g., Huy, Ngoc). The motivation to reconnect with parents, spend time with them, and forgive them for being strict in the past emerged after they developed a thorough understanding of themselves in their relationship with their parents, about the family’s history, and about why their parents did what they did, that is, when their comprehensibility was enhanced, as defined by salutogenesis theory. However, it could also be reasonable to argue that, for these Vietnamese children, reconnecting with family is always an essential thing that adult children wish to do, which is why they could not just erase their ties with their parents and feel at ease, even when they were staying apart from their family. Their motivation (meaningfulness) was always there; they just did not know how to do it yet, until their comprehensibility of themselves and their relationship with their parents improved. This could be a good point. However, if the essence of meaningfulness is defined as one’s active participation in finding a challenge, though painful, worthy of coping with, it is not clear in this study that meaningfulness comes first in this process. Instead, some adult children (Huy, Ngoc, Hau) chose to avoid their parents, a perceived source of intensive stressors, and they played an active role in reconnecting with their parents after their understanding of their situation was improved, so that stressors (the parents) were then perceived as non-stressors.

Comprehensibility, as a SOC component among the adult children of Vietnamese boat refugees who have successfully overcome the intergenerational impact of trauma, covers multiple aspects. It includes the children’s understanding of their family’s migration-related trauma, how these traumatic experiences influenced the parents’ interactions with their children, what the language of love of their refugee parents truly is, why their parents did what they did in their new country, and what the good sides of their families and home culture are. This understanding could emerge through the children’s own reflections on their life experiences (e.g., Ngoc’s narrative) or through professional or external support (e.g., psychologists and teachers in Huy’s and Hieu’s accounts). A thorough understanding of their own family and culture then plays an important role in children of Vietnamese boat refugees creating meaning for themselves and their own lives and in transforming their relationship with their parents, which then contributes to their health and well-being. This is aligned with existing psychological intervention approaches for trauma-impacted families [71,72]—enhancing the ability to interpret thoughts and feelings related to trauma and to make meaning of one’s own experiences.

While the study was successful in obtaining the rich lived experiences of the children of Vietnamese boat refugees, the small number of participants and the lack of gender balance might limit the patterns of themes to some extent. The initial study design with diverse types of interview participants (the adult children and the religious leaders) for triangulation [73] has not yet been successfully applied for this topic, intergenerational trauma, where its manifestations often occur within family interactions and might not be obvious to outside observers, such as religious leaders. This methodological point should be considered in future research designs. Also, compared to the other adult children, the negative impact of intergenerational trauma experienced by Hung seemed to be slightly less intense, suggesting that not all the trauma-exposed parents had a similar level of negative impacts on their children. This is possibly due to the parents’ own recovery and resilience, which is not confirmed through this study, and could be promising for future research, e.g., intergenerational resilience. A key strength of this study is the combination of the concept of the differentiated self from the Bowen family systems theory [59,60] and the sense of coherence concept from salutogenesis theory [61,62] to address a possible solution and pathways to overcome the intergenerational impact of trauma. This is especially meaningful for those who have been affected by trauma in moving forward with coherence, meaning, and health. This also contributes to the current intervention approach for trauma-affected families by shifting the focus from childhood intervention for intergenerational trauma [15] to adult meaning-making and differentiation from maladaptive family impacts. The hybrid analytic method is another strength of this study. The deductive part informs well-framed findings; meanwhile, the inductive part generates insights from rich narratives to seek a relevant conceptual framing. This hybrid analysis process, with iterations between data and conceptual framing, strengthens the credibility of this research.

This study also suggests the need for healthcare support (e.g., screening to discover need, workshops, and dialogue for reflection) among the adult children of refugees to help them better understand themselves in relation to their home culture and their family’s history of trauma as a pathway to grow beyond intergenerational trauma. Healthcare professionals who support refugees should be aware that while the transmission of intergenerational trauma is shared across different communities, their expressions might be slightly moulded by the culture of the population. Accordingly, healthcare services (e.g., counselling, family interventions, and community-based interventions) for refugee populations should be provided in a culturally responsive and adaptive manner. Also, in supporting the adult children of refugees, regardless of how painful their experiences have been, it is very important to help them find meaning in these experiences. Without seeing any good sides to their situation, without finding any meaning in their childhood maladaptive family interactions, and without realizing that their parents actually care for them in some ways, they might not ever feel calm and at ease about their family of origin or their past. Of course, it would be ideal if the parents could change or if the parent–child relationship could be improved, but this might not always be the case for adult children, especially if their parents have died before this could happen.

## 7. Conclusions

This study extends the literature on intergenerational trauma by centering the voices of adult children of Vietnamese boat refugees and illuminating how individuals move forward to health despite enduring trauma-shaped family interactions. While grounded in the specific historical and cultural context of the Vietnamese forced migration, the findings offer transferable insights into how intergenerational trauma is sustained and transformed across diverse cultural settings. Across the narratives, intergenerational trauma was transmitted not solely through recounting parents’ migration-related trauma, but also through trauma-shaped daily child–parent interactions, including harsh parenting, high achievement expectations, obligation to compensate parents, and adultification. These mechanisms echo patterns documented among other refugees, as well as Indigenous, Holocaust-surviving, and conflict-affected populations, suggesting that while the expressions of intergenerational trauma are culturally situated, the mechanisms of trauma transmission are structurally consistent across contexts.

Critically, this study shifts the attention toward a less-examined but highly consequential population: adult children who no longer reside with their family of origin yet who continue to carry the emotional and relational legacies of trauma sustained through maladaptive interactions with parents in childhood. The findings suggest that recovery in adulthood from the intergenerational impact of trauma is not about erasing family ties, but rather playing an active role in reframing them through enhanced understanding, applying meaning-making to their past. This is how the self-differentiation from the impact of maladaptive patterns in their family of origin occurs. This is also a sense-of-coherence-enhancing process for better health and well-being, which is facilitated by generalized resistance resources.

## Figures and Tables

**Table 1:** List of participants.

	No.	Pseudonym	Sex	Age When Parents Fled Vietnam
**Group 1** (The children of Vietnamese boat refugees)	1	Hung	M	Not yet born
	2	Huy	M	Three months old
	3	Hieu	M	Not yet born
	4	Hau	M	Not yet born
	5	Ngoc	F	Six months old
	6	Nhi	F	Five years old
**Group 2** (Religious leaders)	7	R1	M	Not applicable
	8	R2	M	
	9	R3	M	
	10	R4	M	

**Table 2:** Global Theme 1—Maladaptive Parent–Child Interactions as Manifestations of Intergenerational Impacts of Trauma in Families.

Codes	Basic Themes	Organizing Themes
Expect children to focus on studying;	1. Poor parenting—high expectations and strict discipline	Maladaptive parent–child interactions
Do not allow children to be distracted by personal interests or hobbies;		
Violence.		
Do not want to disappoint family;	2. Compensate parents—please parents, adultification	
Complete the family’s wishes;		
Deal with health insurance, bank account, house contractor, etc.;		
Give up education to share financial stress with parents;		
Do not want to disappoint family.		
Family is a bad, chaotic place;	3. About self in relation to parents	Children’s emotional confusion in childhood families
Parents’ expectations are weird and unfair;		
Whatever I did, I was never enough for my father.		
I am not as good as my family believes;	4. About self	
If I could come back, I would not give up my education.		

**Table 3:** Global Theme 2—A Strong Sense of Soherence as a Pathway to Outgrow the Intergenerational Impacts of Trauma in Families.

Codes	Basic Themes	Organizing Themes
Physical distance for avoidance;	1. Temporary distance from family impacts in adulthood to sort out	Opportunities and resources facilitate the development of a differentiated self
Distance thanks to the natural transition to adulthood.		
Teachers, friends;	2. Social support	
Siblings.		
Psychologist;	3. Healthcare service	
Psychological confrontation, reflection.		
Parents’ regret of being harsh;	4. Parents change	
Parents tried to connect with children.		
They suffer from a big loss, that’s why they impose high expectations;	5. Among children who avoid parents due to their poor parenting	Enhancing understanding of self in relation to families
How my father treated me impacts how I treated others—I could not control my anger.		
I carried my family’s expectations, which cost me a lot;	6. Among children who lost themselves when pleasing or supporting family	
I wish I could have continued my higher education.		
Follow true career calling;	7. Gain an independent self to direct one’s own life and choices	Reframe the position of self in relation to families
Now my youngest sister supports my parents.		
Forgive parents;	8. Reconnection with parents	
Appreciation of parental language of love.		
From hatred and anger to feeling calm about parents;	9. Peace and Calm	Experience family as a source of ease and inspiration
I’ve had a good family.		
My parents started everything from zero, so I could;	10. Appreciation and inspiration	
Thanks to my parents, I have a good education, and I am here.		

## Data Availability

The data are not publicly available due to privacy and ethical restrictions.

## Abbreviations

The following abbreviations are used in this manuscript:

SOC	Sense of Coherence
GRRs	Generalized Resistance Resources
